# Systematic identification of cell-cell interactions associated with the severity of patients with Alzheimer's disease

**DOI:** 10.1177/13872877261441603

**Published:** 2026-04-15

**Authors:** Zijian Zhang, Naail Chowdhury, Jing Dong, Lang Wu, Chao Cheng

**Affiliations:** 1 Section of Epidemiology and Population Science, Department of Medicine, 3989 Baylor College of Medicine, Houston, TX, USA; 2 Dan L. Duncan Comprehensive Cancer Center, 3989 Baylor College of Medicine, Houston, TX, USA; 3 The Institute for Clinical and Translational Research, 3989 Baylor College of Medicine, Houston, TX, USA; 4 Department of Interdisciplinary Oncology and Department of Genetics, LSU-LCMC Health Cancer Center, School of Medicine, Louisiana State University Health Sciences Center, New Orleans, LA, USA

**Keywords:** Alzheimer's disease, biomarkers, cell-cell interactions, transcriptomes

## Abstract

**Background:**

Alzheimer's disease (AD) is a complex, multifactorial neurodegenerative disorder involving dysfunction across multiple brain regions. While accumulating evidence has implicated the roles of diverse cell types, including neurons, glia, and vascular cells in AD pathogenesis, it is still poorly understood how cell type interactions drive or respond to the disease progression and severity.

**Objective:**

This study aimed to systematically characterize cell-type–specific alterations and intercellular communication changes associated with AD progression.

**Methods:**

We leveraged the transcriptome profiling and a previously established statistical framework to present a comprehensive mapping of the cellular interaction landscape in the human brain of AD.

**Results:**

We identified a wide array of AD-associated cell-cell interactions (CCIs), including not only between the non-neuronal and neuron cells, but also among different neuron subtypes. These patterns were further supported by cell-type signature scoring. Moreover, due to the flexibility of the framework, we further examined CCIs associated with clinical dementia rating across multiple cortical regions. Our findings revealed that the temporal and frontal cortices showed a stronger correlation with dementia severity. However, the subregions of the temporal area show specific dementia-associated CCIs, especially between the inferior and middle temporal gyrus.

**Conclusions:**

Our work advances our understanding of the cellular microenvironment in AD, offering novel insights into how intercellular interactions shape disease trajectory and cognitive outcomes.

## Introduction

Alzheimer's disease (AD) is the most common neurodegenerative disorder and dementia-causing disease, affecting millions worldwide and imposing profound personal and societal burdens.^1^ Pathologically, AD is marked by extracellular amyloid-β (Aβ) plaque accumulation and intracellular neurofibrillary tangles of hyperphosphorylated tau.^2^ These pathological changes disrupt neuronal functions and lead to the death of neurons, ultimately causing the symptoms of memory loss, cognitive decline, and behavioral changes. Although neuronal dysfunction and loss have historically taken center stage in AD research, there is growing recognition that non-neuronal cells—particularly microglia, astrocytes, and oligodendrocytes—play pivotal roles in disease initiation and progression.^3–5^ For example, microglia can adopt disease-associated phenotypes that influence plaque dynamics,^6^ while reactive astrocytes may shift toward neurotoxic or neuroprotective states depending on local cues.^7^ In parallel, oligodendrocyte dysfunction^8^ and white matter disruption^9^ are increasingly linked to AD pathology, suggesting that a broader cellular landscape underlies disease progression beyond purely neuronal-centric mechanisms.

AD progresses across multiple brain regions from the entorhinal cortex, hippocampus, and thalamus, and finally, the neocortex. In the cortex, the front and temporal lobes are most involved. Single-cell or single-nucleus RNA sequencing studies for one specific region or multiple brain regions revealed complex networks of cell–cell communication in AD. They discovered distinct glial subpopulations emerge in early versus late stages of disease,^10^ crosstalk between microglia and astrocytes regulates cytokine signaling and synaptic pruning,^11^ and vascular and immune cells may modulate blood–brain barrier integrity and peripheral immune cell infiltration.^12^

Despite the valuable insights gained from single-cell transcriptome data, their capacity to decipher cell-cell interactions remains limited. Most computational frameworks for inferring the intercellular network rely on the co-expression of ligand-receptor gene pairs.^13^ Such approaches are not well-suited to characterize neuron-to-neuron communications because signaling between neurons is mediated by neurotransmitters, typically small molecules such as glutamate and gamma-aminobutyric acid (GABA). In addition, single-cell approaches remain prohibitively expensive and technically challenging for large cohorts or longitudinal sampling. High per-sample costs, variable tissue quality, and complex library preparation pipelines limit its scalability. In contrast, there are numerous publicly available bulk transcriptomic datasets offering extensive coverage across brain regions and patient populations.

In this study, we leveraged these bulk RNA-sequencing repositories alongside an established statistical framework, previously applied to characterize the tumor microenvironment.^14^ We adapted this approach to analyze the bulk transcriptome profiles from human postmortem brain samples, enabling us to systematically mapping of interactions between neurons and non-neuronal cells, as well as among neurons, in the context of AD.

## Methods

### Gene expression data used in this study

In this study, we analyzed three independent AD gene expression datasets generated using various platforms from previously published studies^15–17^. All datasets were downloaded from the Gene Expression Omnibus (GEO) and provided normalized expression profiles at the probeset level.^18^ Probeset values were converted to gene-level expression using platform annotations from the corresponding GEO platform (GPL) files. For genes with multiple probesets, we used the average expression for two-channel microarrays, while for one-channel microarrays, we selected the probeset with the highest hybridization intensity across samples to represent the gene expression. The following AD datasets were included in this analysis:

GSE132903: This dataset includes gene expression profiles for 97 AD cases and 98 healthy controls, generated using the one-channel Illumina Human HT-12 v4 microarray platform. Samples were collected from the middle temporal gyrus.^15^

GSE95587: This dataset provides RNA-seq data for 84 AD cases and 33 neurologically normal controls, with corresponding Braak staging scores. Samples were collected from the fusiform gyrus and were sequenced with Illumina HiSeq 2500.^16^

GSE84422: This dataset includes gene expression profiles for postmortem human multi-region brain samples and was profiled using the Affymetrix GeneChip platform.^17^ Samples were collected from 19 different brain regions across varying degrees of AD pathology, including 690 definite AD samples, 383 probable AD samples, 475 Possible AD samples, and 456 Normal samples. For each region, the number of available samples ranges from 50 to 60. Each sample in this dataset is annotated with clinical measurements, including age, sex, race, postmortem interval minutes, and PH. Additionally, it also provides cognitive measurement (Clinical Dementia Rating, CDR) and multiple neuropathological metrics, including Braak Neurofibrillary Tangle Score (Braak), average neurotic plaque density, sum of Consortium Establish a Registry for Alzheimer's Disease (CERAD) rating scores in multiple brain regions, and sum of neurofibrillary tangle density in multiple brain regions.

### Marker genes for brain cell types

Two sets of marker genes were used in this study. The first set provides marker genes for six broad brain cell subtypes: neurons, astrocytes, oligodendrocyte precursor cells (OPCs), oligodendrocytes, microglia, and endothelial cells. These markers were defined by analyzing transcriptome-wide RNA expression datasets from human brains.^19^ The second set includes marker genes for 19 specific brain cell types, comprising five GABAergic neurons (Lamp5, Sst, Pvalb, Vip, Sncg), eight glutamatergic neurons (L2/3IT, L5/6NP, L5ET, L5IT, L6B, L6CT, L6IT, L6ITCar3), and six non-neuronal cell types (microglia/perivascular macrophage (Micro-PVM), astrocyte, oligodendrocyte, endothelial cell, OPC, and vascular leptomeningeal cell (VLMC). These marker genes were obtained from scBrainMap, a comprehensive database integrating 715 publicly available scRNA-seq datasets across species, providing brain cell-type annotations and associated marker genes.^20^ In our study, we selected the cell types from normal human brains and aggregated them into the marker gene sets. The data were downloaded from https://scbrainmap.sysneuro.net/.

### Identification of cell-cell interactions associated with AD

We adapted a previously developed algorithm, TimiGP, originally designed to identify cell–cell interactions (CCIs) associated with cancer prognosis,^14^ to infer CCIs associated with AD. The CCIs identified by this method represent cell-cell pairs with inferred relative abundance being associated with the disease status or other clinically relevant phenotypes, rather than the direct physical cell-cell interactions. The modified pipeline involves the following steps:

#### Step 1: Convert gene expression to marker gene pair (MGP) matrix

Using bulk gene expression profiles from S samples (AD and control), we enumerated all possible MGPs (g_i_→g_j_) formed from M marker genes across all brain cell types. For each sample, we calculated the log ratio of expression (g_i_/g_j_), yielding an MGP matrix of size M*(M-1) X S.

#### Step 2: Identify MGPs with differential log ratios between AD and control samples

For each MGP (row) in the matrix, we compared its log ratios between AD and control samples using a student's t-test to obtain a comprehensive set of significant MGPs. Alternatively, we also used a linear model implement in *limma,* adjusting for the available covariates, to define significant MGPs.

#### Step 3: Construct the directed gene-gene network

Because the gene pairs g_i_→g_j_ and g_j_→g_i_ are reciprocal, we only retained the gene pair with significantly higher log ratios in AD. By connecting all significant gene pairs, we generated a directed gene-gene network, in which nodes represented marker genes and edges indicated elevated log ratios of the two genes in AD.

#### Step 4: Infer the cell-cell interaction network

To determine whether relative abundance between two brain cell types (X→Y) is altered in AD, we examined enrichment of significant MGPs from X-specific to Y-specific marker genes. The expected number of such interactions was calculated as $L_{X}\cdot L_{Y}\cdot f$ , where $L_{X}$ and $L_{Y}$ are the counts of X- and Y-specific markers, and f is the background rate of significant MGPs. Enrichment was assessed via Fisher's exact test. Significant cell-type interactions were then integrated into a directed CCI network associated with AD.

This framework was also applied to identify CCIs associated with AD severity/Clinical Dementia Rating. For these outcomes, in Step 2, MGP log ratios were tested for correlation with severity using Spearman correlation.

### Calculation of the cell signature scores

We applied the BASE algorithm to calculate signature scores for each brain cell type in each sample based on gene expression profiles.^21^ This algorithm employs a rank-based approach to assess the expression levels of marker genes corresponding to a specific cell type, generating a sample-specific score. A higher score indicates that the marker genes are more highly expressed in a given sample, suggesting a greater relative abundance of that cell type. Additional methodological details about the BASE algorithm can be found in a previous study.^22^

### Other bioinformatics and statistical analysis

Differential gene expression analysis between AD and control was performed with a student's t-test or linear model implemented in *limma* for each gene. Multiple testing correction was applied using the Benjamini–Hochberg method to yield false discovery rates (FDR). Pearson and Spearman correlation coefficients were used to quantify and estimate the statistical significance of the associations between continuous variables.

All analyses were conducted using the R programming environment (version 4.1.0). Data wrangling and visualization were performed using a suite of R packages. Data manipulation was facilitated by dplyr (v1.0.7), reshape (v0.8.8), and data.table (v1.14.0). Data visualization was carried out using ggplot2 (v3.3.5), gridExtra (v2.3), ggrepel (v0.9.1), ComplexHeatmap (v2.10.0), RColorBrewer (v1.1-2), and circlize (v0.4.13).

## Results

### Computational identification of brain cell type pairs with relative abundance associated with AD

AD is a neurodegenerative disorder characterized by neuronal loss, synaptic dysfunction, and increased neuronal apoptosis.^23^ While the loss of neuron cells contributes to disease development and progression, other non-neuronal (glial) cell types also play critical roles through intercellular interactions. We hypothesize that the relative abundance between specific brain cell type pairs is more strongly associated with AD pathology than alterations in individual cell types alone. To test this hypothesis, we begin our analysis using six major brain cell types in the Piras dataset: neurons, astrocytes, microglia, oligodendrocytes, OPCs, and endothelial cells.

**Figure 1.**
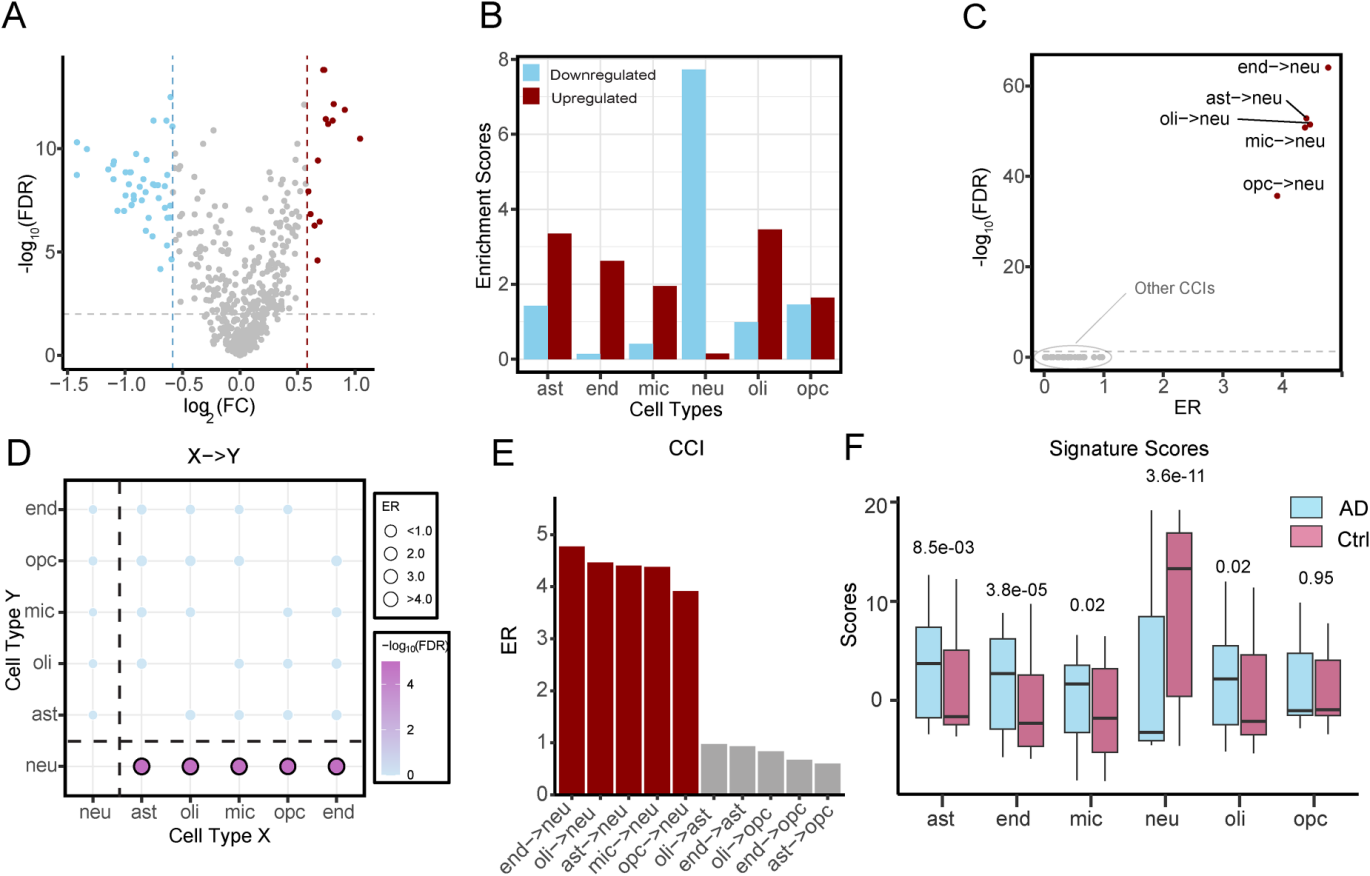
Identification of cell-cell interaction pairs from the human brain transcriptome. (A) Differentially expressed genes between Alzheimer's disease (AD) patients and health controls. Dashed lines denote significance thresholds (|FC| > 1.5, FDR < 0.05). Upregulated genes are shown in red, and downregulated genes in blue. (B) Enrichment analysis of the predefined marker genes across six major cell types for both upregulated and downregulated genes. The enrichment was assessed using a hypergeometric test, and the enrichment score is defined as -log10(p-value). (C) Volcano plot displaying the cell-cell interaction pairs associated with AD. Interactions with FDR < 0.05 are considered significant. (D) Bubble plot displaying all the possible interaction networks, with bubble size and color representing the enrichment ratio (ER) and q-value. (E) ER of the top ten cell-cell interactions, highlighting the intensive interaction between non-neuron cell types with neurons. Red represents the interactions with FDR < 0.05. (F) Signature scores of each cell type in the AD and control groups, calculated based on the predefined marker genes. ast: astrocytes; end: endothelial cells; mic: microglia; neu: neurons; oli: oligodendrocyte cells; opc: oligodendrocyte precursor cells.

The Piras dataset provides microarray gene expression profiles from middle temporal gyrus samples of 97 AD patients and 98 healthy controls.^15^ Differential expression analysis identified 2894 genes significantly dysregulated in AD at FDR < 0.05, including 1432 upregulated and 1462 downregulated genes (Figure 1A). As expected, we observed strong enrichment of neuronal marker genes among downregulated genes (7.7-fold), whereas markers of non-neuronal cells were enriched among upregulated genes (Figure 1B).

To evaluate how the relative abundance between brain cell type pairs is associated with AD, we applied the cell-cell interaction analysis described in the Methods section. This framework identifies AD-associated CCIs by assessing the enrichment of cross-cell-type marker gene pairs within the set of AD-associated gene pairs. For each directed cell pair, we calculated an enrichment ratio (ER) and estimated statistical significance using a permutation-based test. In the Piras dataset, five out of 30 possible directed cell pairs were significantly associated with AD (FDR < 0.05) (Figure 1C, D). These significant CCIs included endothelial cells → neurons (end→neu), astrocytes → neurons (ast→neu), oligodendrocytes → neurons (oli→neu), microglia → neurons (mic→neu), and oligodendrocyte precursor cells → neurons (opc→neu). The enrichment ratios of these CCIs were higher than those with non-significance (Figure 1E). These results indicate that the relative abundance of multiple non-neuronal cell types versus neurons is significantly elevated in AD compared with control samples.

Further, we assessed relative cell-type abundance using marker gene signature enrichment analysis, which is a more robust method and less susceptible to noise in low-abundance cell populations. This analysis confirmed a reduced abundance of neurons and increased enrichment of astrocytes, endothelial cells, microglia, and oligodendrocytes in AD samples (Figure 1F). These findings align with previous studies emphasizing the critical role of cell-type composition in AD pathophysiology. Notably, the loss of neurons and abnormal increase in glial populations, such as microglia, astrocytes and oligodendrocytes, have been recognized as hallmark features of AD progression.^24^

### Identification of AD-associated CCIs across 19 distinct human brain cell types

**Figure 2.**
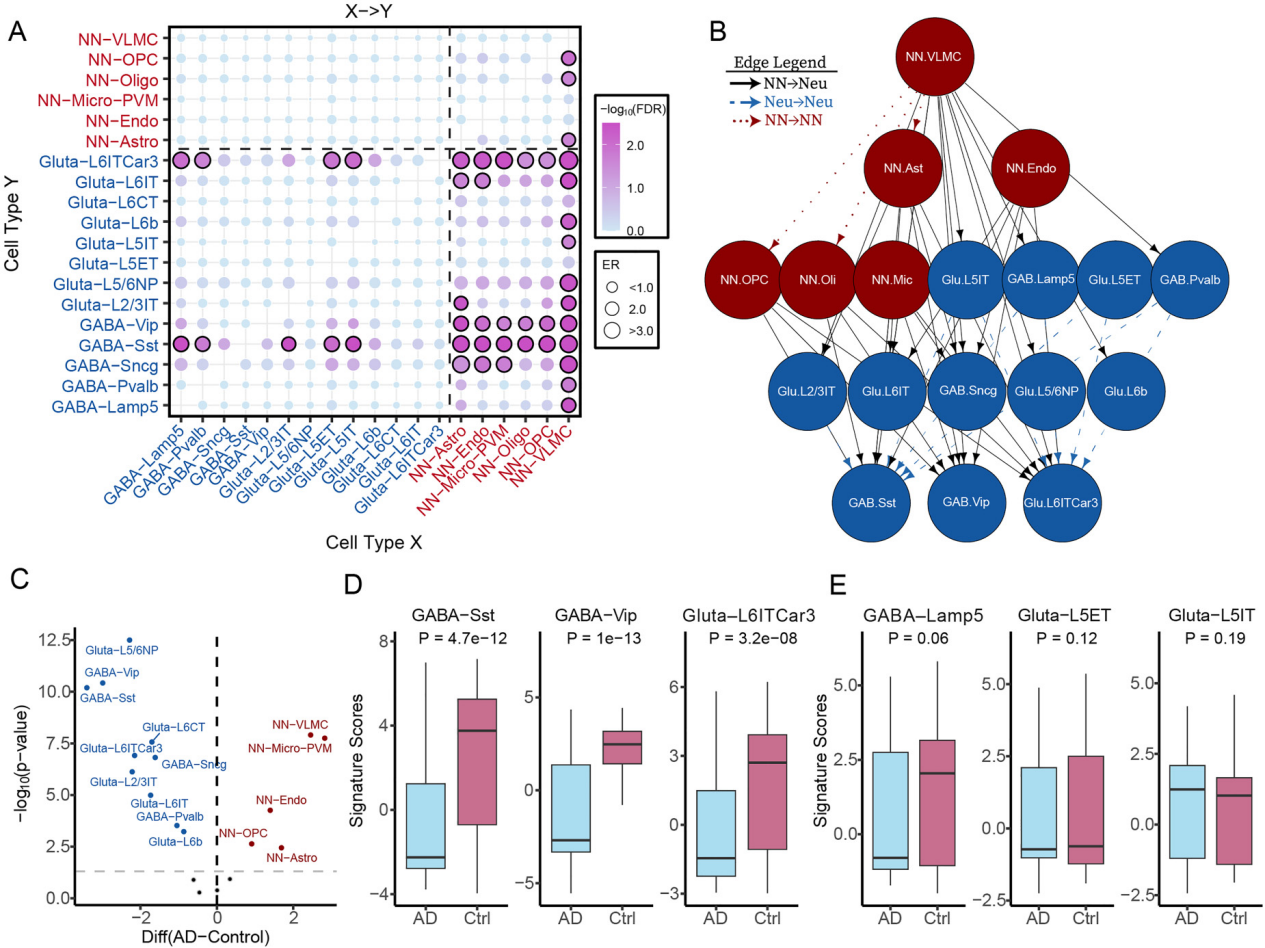
Comprehensive mapping of intercellular interactions across 19 distinct brain cell populations. (A) Bubble plot of predicted cell–cell interactions (CCIs) among 19 cell types, including diverse neuronal subtypes. The source (sender) cell types are on the x-axis, and target (receiver) cell types are on the y-axis. Bubble size represents the enrichment ratio (ER) of each interaction, while color-coding indicates statistical significance (FDR). (B) Network diagram of significant CCIs (FDR < 0.01), highlighting cell types with high in-degree or out-degree connectivity. Nodes are color-coded by broad cell class—GABAergic neurons, glutamatergic neurons, and non-neuronal cells—to visualize interaction dynamics. (C) Volcano plot illustrates the cell-type signature scores between AD and control samples. Each point represents a cell type, with the x-axis showing the difference in mean signature score and the y-axis its statistical significance. (D) Box plots comparing signature scores for three heavily impacted GABAergic neuron subtypes—Sst, Vip, and Glutamatergic L6ITCar3—between AD and control. (E) Box plots for signature scores of less affected neuron types—GABAergic Lamp5and Glutamatergic L5ET and L5IT. NN: Non-neuron; Gluta: Glutamatergic; GABA: GABAergic; VLMC: vascular leptomeningeal cells; Micro-PVM: microglia/Perivascular macrophages; Astro: Astrocytes; oli: oligodendrocyte cells; opc: oligodendrocyte precursor cells; Endo: Endothelial cells.

Having demonstrated the effectiveness of our CCI analysis at the level of six major brain cell classes, we extended the analysis to finer-resolution interactions using 19 cell types defined from scRNA-seq data by Chi et al.^20^ In the 19 cell types, we included five subtypes of GABAergic neurons, eight subtypes of glutamatergic neurons, and vascular leptomeningeal cells (VLMC), which are essential for maintaining the vascular and immunological health of the brain.^25^ In the Piras dataset, we identified 44 significant CCIs (FDR < 0.05) with elevated relative abundance in AD samples versus controls (Figure 2A, Supplemental Table 1). Of these, 32 were interactions between non-neuron and neuron, consistent with neuronal loss being a hallmark of AD. Notably, vascular cells, including VLMC and endothelial cells, and glial cells such as astrocytes and Micro-PVMs, exhibited high connectivity with neuronal subtypes, particularly GABAergic-Sst, GABAergic-Vip, GABAergic-Sncg, and Glutamatergic-L6IT and L6ITCar3 neurons. Additionally, we observed three Vascular → Glial interactions (VLMC → OPC, VLMC → Astro, VLMC → Oligo). Interestingly, nine Neuron → Neuron interactions involving both excitatory and inhibitory subtypes were detected in our analysis. These interactions included: Glutamatergic-L5IT → GABAergic-Sst, GABAergic-Lamp5 → GABAergic-Sst, Glutamatergic-L2/3IT → GABAergic-Sst, Glutamatergic-L5IT → Glutamatergic-L6ITCar3, Glutamatergic-L5ET → GABAergic-Sst, GABAergic-Lamp5 → Glutamatergic-L6ITCar3, Glutamatergic-L5ET → Glutamatergic-L6ITCar3, GABAergic-Pvalb → GABAergic-Sst, and GABAergic-Pvalb → Glutamatergic-L6ITCar3. These interactions suggest that distinct neuronal subtypes are differentially impacted during AD progression. We integrated all significant CCIs into a directed hierarchical network based on in-degree and out-degree metrics of each cell type (Figure 2B). VLMCs emerged as the top node, involved in 11 VLMC→Neuron interactions and 3 VLMC →Glial interactions, underscoring their potential role in shaping the brain microenvironment during AD. In contrast, three neuronal subtypes (GABAergic-Sst, GABAergic-Vip, and Glutamatergic-L6ITCar3) resided at the bottom of the hierarchy, indicating their higher vulnerability to AD-associated disease progression.

To support the CCI-based findings, we computed cell-type–specific signature scores for all 19 cell types using marker gene enrichment analysis. Among these, 15 cell types showed significant abundance differences between AD and control samples (p-value < 0.01, Wilcoxon test; Figure 2C). Of the 13 neuronal subtypes, 10 exhibited significantly reduced scores in AD, including GABAergic-Sst, GABAergic-Vip, and Glutamatergic-L6ITCar3 (Figure 2D). No significant changes were observed for Glutamatergic-L5IT, GABAergic-Lamp5, or Glutamatergic-L5ET (Figure 2E). Conversely, five of six non-neuronal subtypes except oligodendrocytes, showed significantly elevated scores in AD, with VLMCs showing the most pronounced increase. These findings validate the CCI-derived interactions and highlight the distinct and potentially divergent roles of neuronal and non-neuronal cells in AD pathogenesis. Collectively, our results demonstrate that CCI analysis can reveal mechanistic insights into the cellular dynamics underlying AD development.

### Validation of AD-associated CCIs using the Freidman dataset

**Figure 3.**
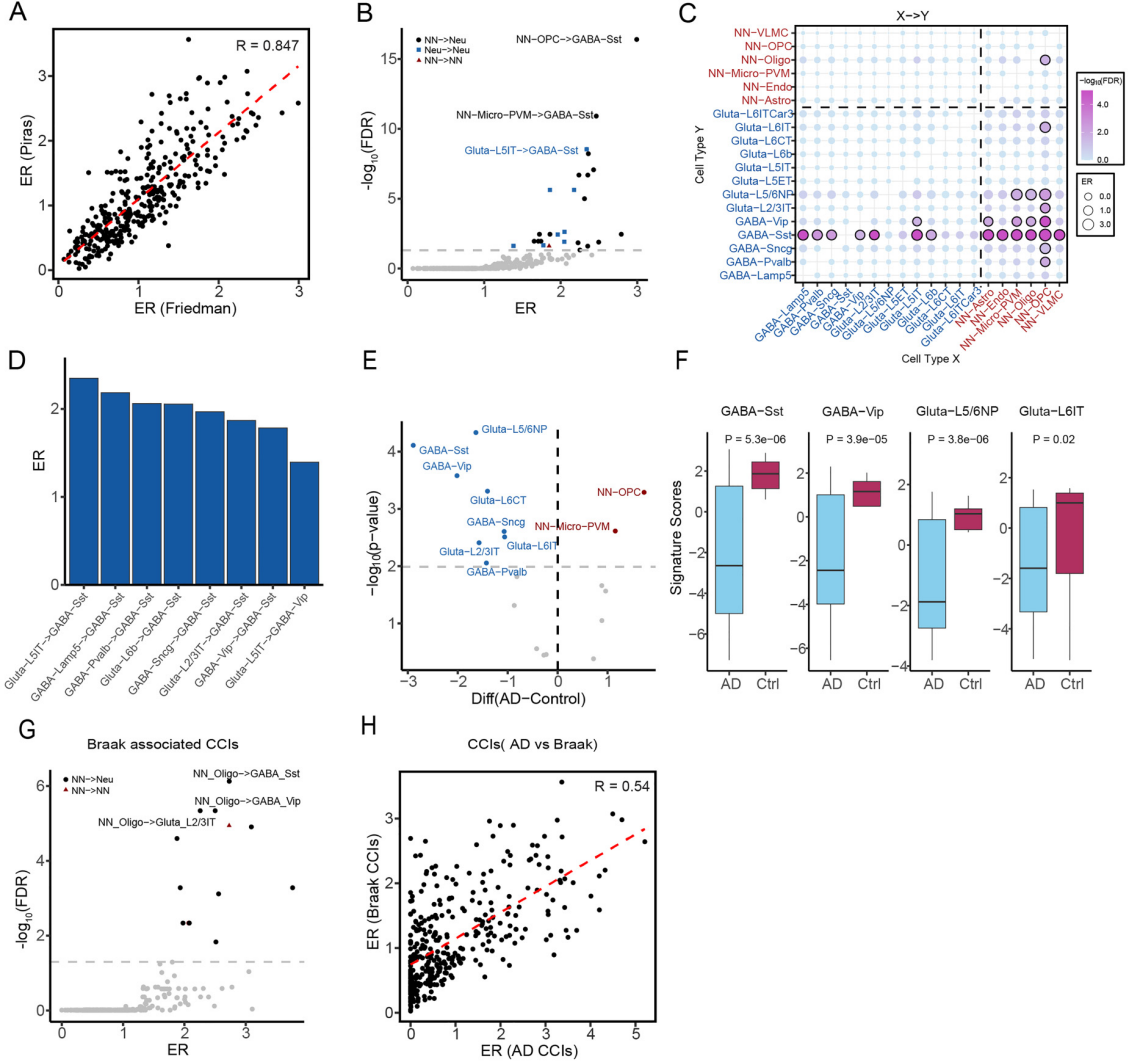
Validation of Alzheimer's-associated cell-cell interactions with the friedman dataset. (A) Correlations of enrichment ratio (ER) of AD-associated cell-cell interactions (CCIs) between Piras and Friedman datasets, showing reproducibility across cohorts. (B) Volcano plot of CCIs detected in the Friedman dataset, with interactions of FDR < 0.05 marked as significant. Black/Circle: CCIs between non-neuron and neuron; Blue/Square: CCIs within neurons; Red/Triangle: CCIs within non-neurons. (C) Bubble chart depicting predicted CCIs in Friedman dataset: sender cell types on the x-axis, and receiver types on the y-axis; dot size indicates ER, and color shows FDR significance. (D) Enrichment ratio of the significant neuron-neuron interactions in the Friedman data, highlighting the intensive interaction between neuron cell subtypes. (E) Signature scores across 19 cell types comparing AD versus control samples using predefined marker genes. (F) Box plots comparing signature scores for four neuronal subtypes heavily impacted GABAergic neuron subtypes—Sst, and Vip, and Glutamatergic L5/6NP, and L6IT—between AD and control individuals. (G) Identification of CCIs correlated with Braak stage, highlighting interactions that track disease progression. Black/Circle: CCI between non-neuron and neuron; Red/Triangle: CCI within non-neurons. (H) Pairwise correlation between ERs of Braak-associated interactions and AD-associated interactions, indicating considerable overlap in signaling alterations.

To validate the CCI findings from the Piras dataset, we performed a 19-brain-cell-type CCI analysis using an independent AD dataset generated by Friedman et al.^16^ This dataset includes RNA-seq profiles from fusiform gyrus samples of 84 AD patients and 33 controls. For each of the 342 directed cell-type pairs among the 19 brain cell types, we calculated enrichment ratios using the CCI framework. To assess consistency across datasets, we computed the Spearman correlation coefficient (SCC) between enrichment ratios from the Piras and Friedman datasets. As shown in Figure 3A, enrichment ratios were highly correlated (SCC = 0.847), indicating strong concordance in AD-associated CCIs between the two datasets.

We identified 26 significant AD-associated CCIs in the Friedman dataset (FDR < 0.05; Figure 3B, Supplemental Table 2). Among them, 17 were non-Neuron→Neuron interactions, 8 were Neuron→Neuron, and 1 was Glial→Glial (Figure 3C). Notably, 14 of these CCIs were also found to be significant in the Piras dataset, supporting the reproducibility of the findings. In particular, 4 of the 8 Neuron→Neuron interactions were consistently identified in both datasets. Among these overlapping interactions, Glutamatergic-L5IT → GABAergic-Sst, GABAergic-Lamp5 → GABAergic-Sst, and GABAergic-Pvalb → GABAergic-Sst were the top three most significant Neuron→Neuron interactions in the Friedman dataset. (Figure 3D).

Among the 17 non-Neuron→Neuron interactions, 10 overlapped with those from Piras. However, the most enriched non-neuronal cell type differed between datasets. In Friedman, OPCs emerged as the dominant top-layer nodes in the CCI network hierarchy (Supplemental Figure 1), contrasting with VLMC cells in the Piras dataset. This discrepancy may reflect regional differences in the vulnerability to AD pathology. Samples in Piras were derived from the middle temporal gyrus, while Friedman samples were from the fusiform gyrus, highlighting region-specific CCI patterns with conserved neuronal targets.

To validate the AD-associated CCI, the cell-type signature scores were compared between AD and control samples. As expected, the neuron cell type scores decreased while the non-neuron increased. Notably, 10 cell types showed significant differences (p-value < 0.01, Figure 3E). Eight neuronal subtypes had reduced scores in AD, including the 4 most vulnerable neuronal subtypes (GABAergic-Sst, GABAergic-Vip, Glutamatergic-L6IT, and Glutamatergic-L5/6NP) discovered in the CCI analysis (Figure 3F). Inversely, no significant alterations were detected in the cell types with less effect on the CCIs (Supplemental Figure 2). Secondly, elevated scores of OPCs and Micro-PVM in AD samples were observed in both datasets. OPCs showed the most pronounced increase, which is consistent with the outdegree analysis from the CCI results (Figure 3E, Supplemental Figure 1). Lastly, although both datasets consistently identified three unaffected cell types from the signature prediction, like Glutamatergic-L5IT, and GABAergic-Lamp5, Oligodendrocytes, their significant interactions with GABAergic-Sst were detected by the CCI analysis (Supplemental Tables 1 and 2).

Braak scores refer to a widely used neuropathological system for classifying the progression of tau pathology, specifically, neurofibrillary tangles.^26^ Given the availability in the Friedman dataset, we extended our application to identify CCIs associated with Braak scores (see Methods). We identified 13 significant CCIs (FDR < 0.05; Figure 3G, Supplemental Table 3). These Braak-associated CCIs showed high concordance with AD diagnosis-based CCIs, as evidenced by a strong correlation in enrichment ratios (SCC = 0.54; Figure 3H). Importantly, the bottom four neuronal nodes associated with AD versus control analysis were also implicated in the Braak-based CCI analysis, reinforcing their relevance in distinct aspects of AD pathology (Supplemental Figure 3). Consistently, OPCs and oligodendrocytes emerged as the most involved cell types in AD and Braak-associated CCIs, respectively. OPCs are the progenitors of oligodendrocytes, and both cell types are closely associated with inflammatory pathology and the toxicity of Aβ.^10^

Still, notable differences were observed between the two sets, potentially reflecting a discrepancy in clinical and pathological manifestations of AD. Most Braak CCIs are between non-neuronal cell types and neurons (12/13), and no interactions between neuronal subtypes (Supplemental Table 3). Eight CCIs between neuronal subtypes were involved in the AD versus control comparison (Supplemental Table 2). This indicated that the Braak scores may not fully capture the complexity of disease phenotypes, which include both cognitive and pathological dimensions.

### CCIs associated with clinical dementia rating in distinct brain regions

Previous studies have demonstrated that AD's progression is marked by distinct regional differences in brain structure and function.^27^ In the early stages, significant damage occurs in the entorhinal cortex and hippocampus, where neurofibrillary tangles and synaptic loss first emerge. As the disease advances, cortical regions, especially those involved in higher cognitive functions, suffer atrophy and hypometabolism that closely correlate with clinical decline. Importantly, this cortical degeneration is not uniform, and it follows a predictable spatiotemporal pattern of regional vulnerability. To characterize these regional differences, we leveraged CCIs to analyze the Wang dataset, which provided CDR and gene expression profiles of 19 brain regions from 118 human brains.^17^ The CDR is a global measure of cognition and functional performance to assess and stage dementia. By correlating CCI with CDR, we characterized the specific cell-cell interaction in the different cortical regions

**Figure 4.**
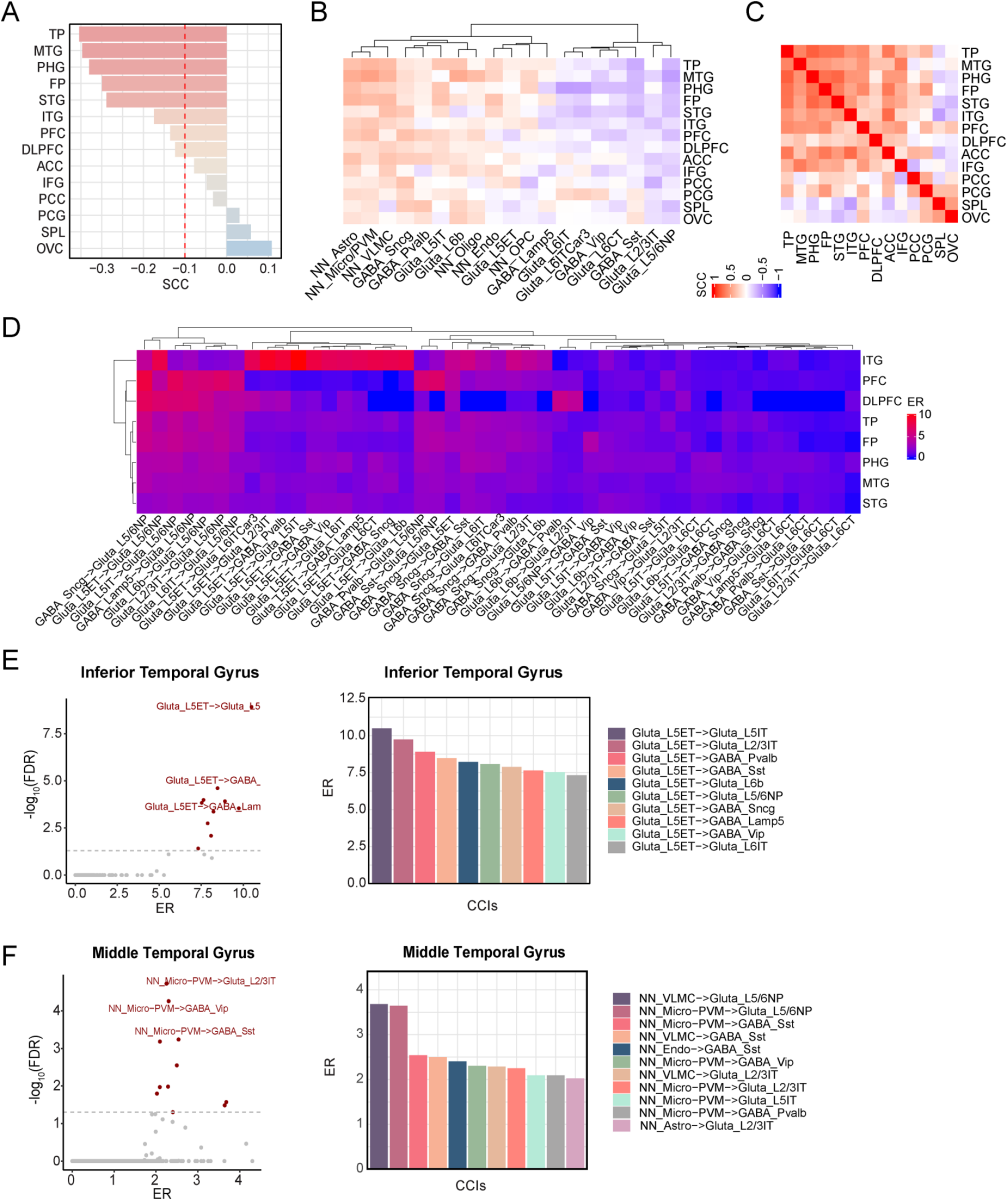
Cell-cell interactions linked to clinical dementia rating (CDR) across multiple brain regions. (A) Barplot illustrating correlations between neuronal cell-type signature and CDR across 14 cortex regions, highlighting areas most associated with dementia severity. (B) Heatmap of the association of 19-cell type signature score versus CDR in the 14 cortex regions, demonstrating the variation of different cell subtypes with CDR. (C) Heatmap displaying the correlation matrix of CDR-associated CCIs across 14 regions. The enrichment ratios across 101 cell-cell pairs (ER > 1.5 and p-value < 0.001 in at least one region) were used to determine the association. (D) Heatmap of ER with hierarchical clustering to reveal patterns of cell-cell signaling across the CDR strongly associated areas. (E) In the inferior temporal gyrus: (left) volcano plot highlighting CCIs associated with CDR; significant interactions (FDR< 0.05) are marked as red, and (right) bar graph of enrichment ratios for the top significant interactions. (F) Same analysis as (E) but performed on the middle temporal gyrus. TP: Temporal Pole; MTG: Middle Temporal Gyrus; PHG: Parahippocampal Gyrus; FP: Frontal Pole; STG: Superior Temporal Gyrus; ITG: Inferior Temporal Gyrus; PFC: Prefrontal Cortex; DLPFC: Dorsolateral Prefrontal Cortex; ACC: Anterior Cingulate, IFG: Inferior Frontal Gyrus; PCC: Posterior Cingulate Cortex; PCG: Precentral Gyrus; SPL: Superior Parietal Lobule; OVC: Occipital Visual Cortex.

First, we evaluated the relationship of CDR with neuron cell-type signature scores across cortical regions in Wang's dataset (Figure 4A). We found that the temporal (Temporal Pole: TP, Middle Temporal Gyrus: MTG, Superior Temporal Gyrus: STG, Inferior Temporal Gyrus: ITG) and frontal cortex regions (Frontal Pole: FP, Prefrontal Cortex: PFC, Dorsolateral Prefrontal Cortex DLPFC) exhibited strong correlations with the CDR scale, whereas visual, parietal, and cingulate areas showed weak associations. Next, we expanded this analysis to include the 19 cell types. The resulting correlation heatmap revealed notable differences between the strong and weakly CDR-associated regions (Figure 4B). Even within the regions showing strong global associations, individual neuronal subtypes followed divergent trends, certain show positive associations with dementia severity, others negative associations. These indicated the region-specific complexity of neuronal subtype vulnerability or compensation in AD.

Then we identified the CCIs associated with CDR in each of these regions. For most regions, we identified fewer significant CCIs compared with the Piras and Freidman datasets because of much smaller sample sizes in each region. But we observed that a greater number of significant CCIs (FDR < 0.05) emerged in the temporal and frontal cortices than in other regions (Supplemental Table 4). In Figure 4C, we showed the pattern of 101 CCIs that are significant in at least one brain region (ER > 1.5 and p value < 0.001). As shown, within both the CDR strongly associated regions and weakly-associated regions, the global CCI patterns were highly consistent. In contrast, when comparisons between the strong and weak groups revealed a striking inverse correlation, indicating that these groups represent fundamentally distinct patterns of connectivity.

Next, we focused on regions with strong CDR associations—specifically, the temporal and frontal cortices. Further dissection at the level of individual CCIs revealed region-specific interactions (Figure 4D). In the frontal regions (PFC, DLPFC, TP), neuron-neuron interaction patterns were largely consistent across subtypes, suggesting shared signaling alterations. In contrast, temporal regions displayed heterogeneity in ITG and MTG/STG. The ITG exhibited its top ten significant interactions (FDR < 0.05) exclusively among neuronal subtypes, all originating from Glutamatergic-L5ET neurons (Figure 4E). However, in adjacent regions such as the MTG, the most enriched interactions were between non-neuronal and neuronal cells (Figure 4F), indicating that even within the temporal lobe, regional CCIs reflect diverse pathological processes and highlight the complexity of local microenvironments.

## Discussion

In this study, we adopted an established framework to investigate cell-cell interactions in AD. This framework has been developed to study the cell-cell interactions within the tumor microenvironment and identify cancer biomarkers from large cohort transcriptome profiling. In the tumor microenvironment, tumor cells interact with immune cells and stromal cells to form a complex network, driving the tumor initiation, progression, and metastasis.^28^ Neuroinflammation studies have suggested that microglia, astrocytes, and neurons act in a synchronized manner to promote neurodegeneration.^3^ Given their similarity, we applied this framework to AD and control human brain samples to dissect CCI landscapes across cell types in AD pathology. It is important to clarify that the inferred CCIs represent pairs of cell types exhibiting significant differences in relative abundance between AD and control samples; thus, they reflect statistical associations rather than direct physical or ligand–receptor interactions.

Here, we profiled the interaction between the six major cell types in the human brain (neurons, microglia, astrocytes, oligodendrocytes, endothelial cells, and OPCs) with samples from the middle temporal cortex, which is the most affected brain region in AD. All five other cell types interacted significantly with neurons. The higher positive enrichment ratio indicated the relative abundance of the cell-type pair. This is consistent with the result of cell type enrichment/depletion from single-cell RNA-sequencing in AD, the depletion of neurons and enrichment of the other cell types in the late stage of AD.^29^ Next, we included the excitatory and inhibitory neuron subtypes. The single-cell RNA-seq well defines these cell types.^30,31^ With the same dataset, we not only identified the interaction between non-neuronal cells and neuron subtypes, but also the interaction within neuronal subtypes. Surprisingly, the enrichment of these CCIs was consistent with the cell type composition and cell-type signature analysis. Our work bridges the gap between single-cell granularity and large-scale transcriptomic accessibility. This enables us to quantify comprehensive interactions in the context of AD with the bulk transcriptome.

By comparing transcriptomic profiles between AD and control samples, we identified a set of AD-associated interactions among neuronal cell types. These results indicated substantial alterations in neuronal cell-type balance during disease progression. Previous studies have reported that an imbalance between excitatory and inhibitory neurons (E/I imbalance) contributes to cognitive symptoms in AD.^32,33^ Our analysis int this study investigated a finer neuronal subtype resolution and revealed more specific AD-associated interactions involving relative abundance changes not only between excitatory and inhibitory neurons, but also among excitatory subtypes and among inhibitory subtypes. Mechanically, these interactions may reflect differential vulnerability of distinct neuronal cell types to the neurotoxic environment in AD, with some cell populations being more resistant and others more susceptible to disease-related stresses.

The correlation analysis between the Piras (MTG) and Friedman (fusiform gyrus) datasets revealed a high degree of reproducibility in AD-associated CCIs, with a Spearman correlation coefficient of 0.847. Fourteen interactions overlapped between the two datasets, suggesting shared pathological mechanisms in the AD. Among them, two inhibitory interneuron types, GABAergic-Sst and GABAergic-Vip, were identified as favorable in AD brains. Dysfunction of these interneurons disrupts the balance between excitation and inhibition in neural circuits, contributing to cognitive deficits, particularly in memory and learning.^34^ The hierarchical analysis revealed that the leading cell populations differed by brain region: vascular leptomeningeal cells (VLMCs) were predominant in the MTG, whereas OPCs in the fusiform gyrus. VLMCs are specialized fibroblast-like cells located in the meninges, which are adjacent to the MTG but distant from the deeper fusiform gyrus. Anatomical proximity may explain why VLMCs are more readily detected in MTG datasets compared with fusiform samples. However, it should be noted that technical factors, including variability in tissue sampling, may also influence the apparent dominance of specific cell types across different brain regions.

When gene expression data from different brain regions were analyzed and compared, we observed marked regional heterogeneity in the AD-associated CCIs. This variation likely reflects differences in cellular composition and functional specialization across brain regions. For example, in the MTG, VLMCs emerged as predominant cell types in the top CCIs, suggesting potential dysregulation of blood–brain barrier function, immune cell trafficking, and neurovascular interactions in this region, which is closely associated with neurovascular and meningeal interfaces. This observation is consistent with growing evidence implicating cerebrovascular dysfunction and immune-related pathways in Alzheimer's disease pathology.^12^ In contrast, the predominance of OPCs in CCIs identified in the fusiform gyrus may indicate region-specific myelin remodeling, neuroinflammatory responses, or impaired oligodendrocyte maturation. OPC activation has been linked to inflammatory signaling and demyelination in AD,^35^ potentially reflecting localized vulnerability of white matter or axonal integrity in this region.

As described by our methodology, we not only detect the AD-associated CCIs when comparing AD versus control, but also CCIs linked to clinical variables (see Methods). Braak stage, reflecting tau pathology progression and cognitive impairment, was used in the Friedman dataset to uncover the Braak-associated CCIs. These interactions predominantly involve oligodendrocytes/OPCs, and neurons, key players in tau-related mechanisms.^36^ Oligodendrocytes form myelin sheaths, enabling rapid electrical conduction and supporting neuronal metabolism. Pathological tau aggregates both within oligodendrocytes and OPCs, disrupting their function and differentiation.^37,38^ The resultant demyelination and impaired OPC maturation compromise axonal conduction and synaptic plasticity, which in turn precipitates cognitive decline. Oligodendrocytes and their progenitors represent a potent and novel target in combating tau pathology, and possibly broader AD progression.

To bridge disease biology with clinical outcomes, we leveraged the CDR scale—a validated and widely adopted tool for quantifying cognitive and functional impairment in dementia.^39^ By examining CCIs correlated with CDR in different cortical regions, we observed strong region-specificity. Even between the adjacent areas like MTG and ITG, the top CCIs differed significantly. In the MTG, interactions were dominated by microglia and other non-neuronal cell types with neurons. In contrast, the ITG was characterized by neuron-neuron interactions led by glutamatergic-L5ET subtypes, suggesting that MTG's vulnerability may stem from its neuroimmune activation profile, while ITG dysfunction may reflect more direct neuronal circuit disruption.

However, this study has several limitations. First, CCI inference is based on bulk transcriptomic profiling, which is susceptible to biases arising from sample preparation, RNA extraction, and other technical variations. Second, the brain samples used for expression profiling were collected postmortem, and mRNA integrity may be substantially degraded depending on the postmortem interval. Such degradation could further compromise the accuracy of inferred relative cell-type abundances. Furthermore, post-mortem data provide only static snapshots of transcriptome at the time of death and failed to capture the dynamic changes. Future studies incorporating longitudinal transcriptomic and functional analyses could provide deeper insights into early-stage pathology. Such studies could help elucidate the temporal dynamics of cellular interactions and their impact on disease progression. Overall, our study underscores the critical value of cell-cell interaction analyses in understanding AD mechanisms. By identifying distinct cellular interaction networks, such network-driven insights can guide targeted therapeutic strategies, reinforcing the translational potential of our findings.

## Supplemental Material

sj-docx-1-alz-10.1177_13872877261441603 - Supplemental material for Systematic identification of cell-cell interactions associated with the severity of patients with Alzheimer's disease Supplemental material, sj-docx-1-alz-10.1177_13872877261441603 for Systematic identification of cell-cell interactions associated with the severity of patients with Alzheimer's disease by Zijian Zhang, Naail Chowdhury, Jing Dong, Lang Wu and Chao Cheng in Journal of Alzheimer's Disease

sj-xlsx-2-alz-10.1177_13872877261441603 - Supplemental material for Systematic identification of cell-cell interactions associated with the severity of patients with Alzheimer's disease Supplemental material, sj-xlsx-2-alz-10.1177_13872877261441603 for Systematic identification of cell-cell interactions associated with the severity of patients with Alzheimer's disease by Zijian Zhang, Naail Chowdhury, Jing Dong, Lang Wu and Chao Cheng in Journal of Alzheimer's Disease
