# Supplementary material for: Systematic identification of cell-cell interactions associated with the severity of patients with Alzheimer's disease
Source: J Alzheimers Dis. 2026 Apr 15;111(3):1156–67. doi: 10.1177/13872877261441603 (PMC13219764; doi:10.1177/13872877261441603)
Supplement: sj-docx-1-alz-10.1177_13872877261441603 - Supplemental material for Systematic identification of cell-cell interactions associated with the severity of patients with Alzheimer's disease [file sj-docx-1-alz-10.1177_13872877261441603.docx]

**Supplemental Material**

**Systematic identification of cell-cell interactions associated with the severity of patients with Alzheimer's disease**


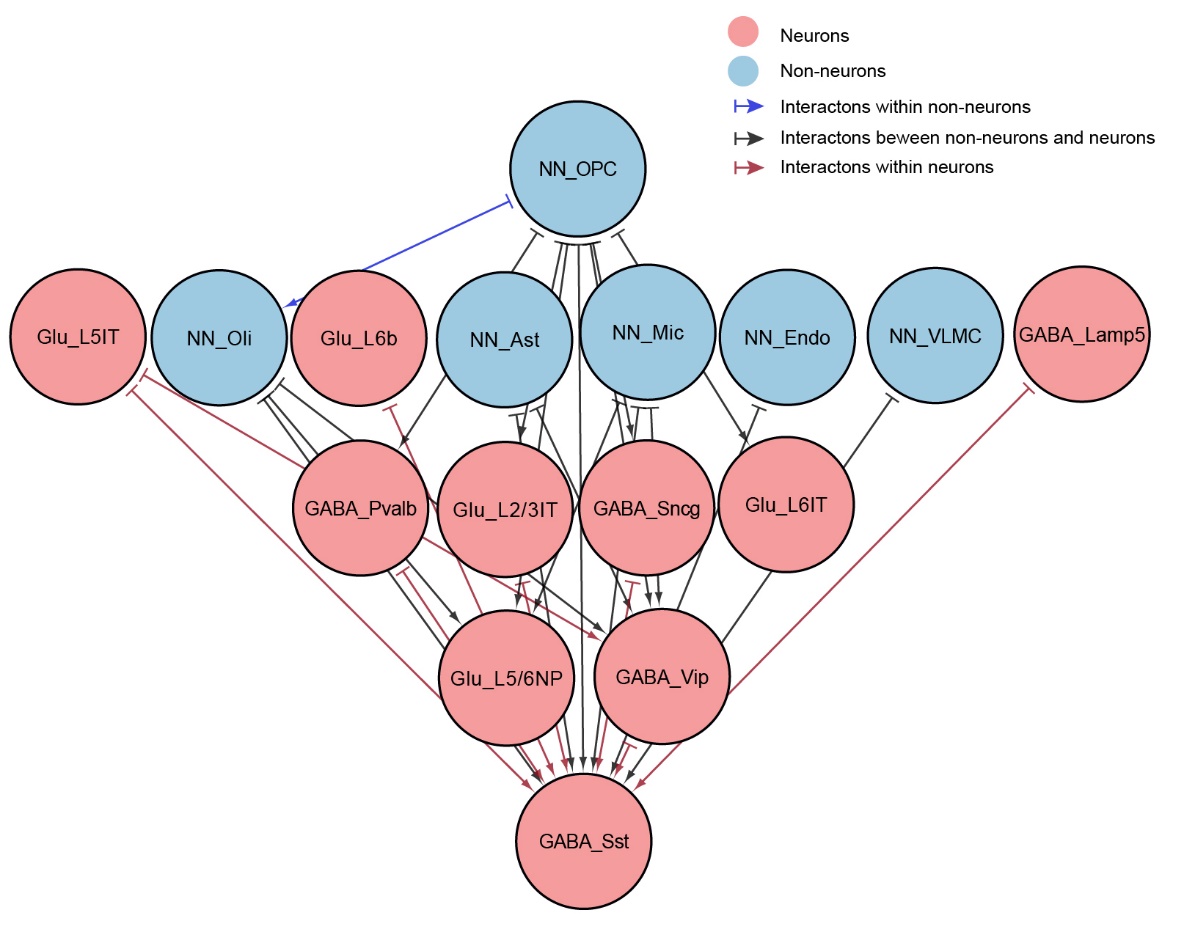


**Supplemental Figure 1.** Hierarchical plot showing the Alzheimer’s disease-associated cell-cell interactions identified from the Friedman dataset.


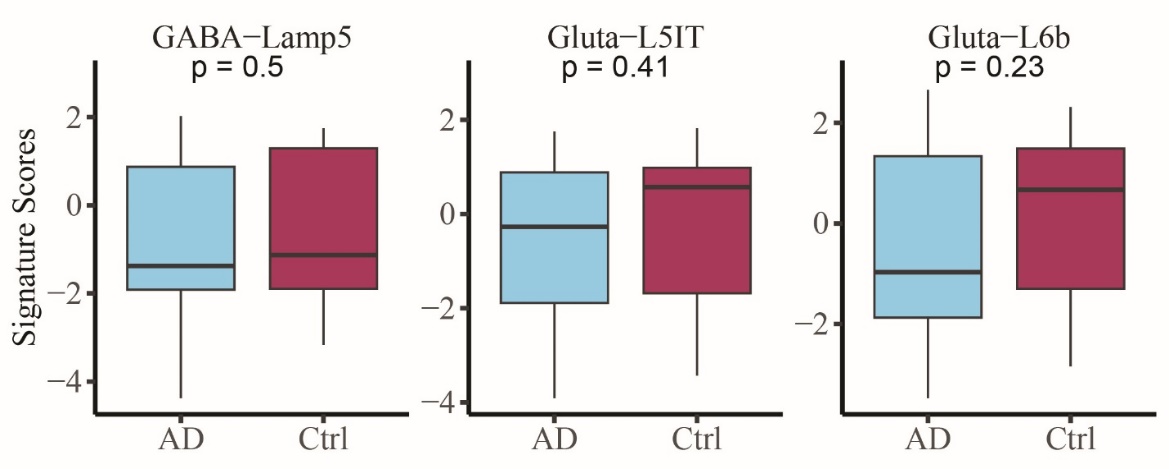


**Supplemental Figure 2.** Cell-type signatures between AD and control in the Friedman dataset.


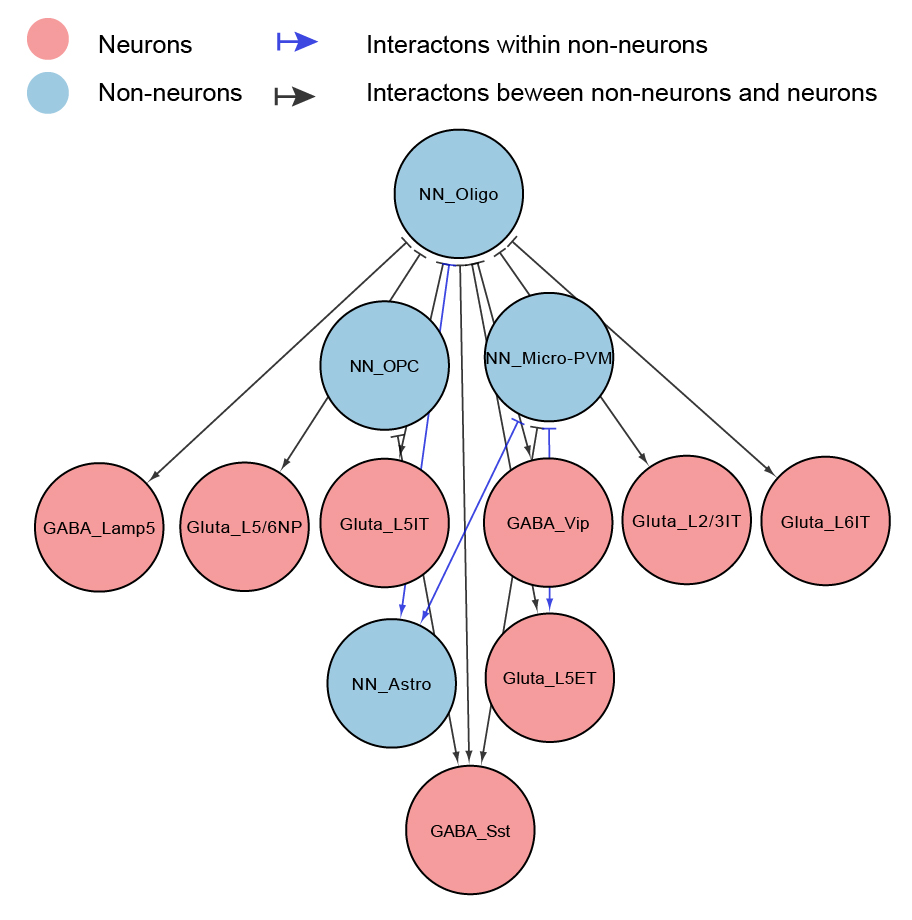


**Supplemental Figure 3.** Hierarchical plot showing the Braak-associated cell-cell interactions identified from the Friedman dataset.
